# On the Surgical Treatment of Croup

**Published:** 1846-10

**Authors:** 


*On the surgical treatment of Croup.* By. M. Guersant, Jun.—I agree in the opinion of MM. Bretonneau, Trousseau, Guersant, Sen., Blache, &c. that to constitute croup, the presence of false membranes in the larynx is essential. The disease may commence at the tonsils, in the bronchi, or suddenly in the larynx itself. In the first case there is more or less redness of the pharynx with swelling of the tonsils, and, what is of vast importance to notice, these last are covered with little white patches, which sometimes extend as far as the velum or uvula.

The *medical* means for treating croup are very limited. *Depletion,* once so freely employed, under the idea that the disease was a simple inflammation, is very rarely of any utility in croup, and oftentimes injurious. *Emetics* have proved far more useful as adjuvants, by favouring the detachment and expulsion of the false membranes; but alone they are not to be relied upon. *Mercurials,* especially when used early, have often exerted excellent effects upon the disease; but for these to be of service the dyspnœa must not be very urgent, or the patient very enfeebled, and when used alone they have effected but few cures.

It is upon surgical treatment we must usually most rely, and by it we mean the *application of caustics to the pharynx,* as well as the operation of tracheotomy. Various fluid or solid substances of this nature have been employed, but we prefer the *nitrate of silver.* Weak solutions suffice at the earliest stage of the disease, when there is little else than the pseudo-membranous deposit upon the pharynx. There are indeed cases in which these are not seen at all, the false membranes being at once deposited in the larynx, but these are rare; and frequently the membranes are not detected in the pharynx, because the first period of the disease has already passed away. At an early period the symptoms are but little urgent, and the physician, little accustomed to treat children, often neglects to examine the throat. With M. G., whenever a child manifests any febrile re-action, *such examination is an invariable rule,* and in this way he has frequently been enabled to detect the approaching disease, which would not otherwise have been suspected. He instances a case in which M. Trousseau was induced fortunately to examine the throat by observing the surface of a blister to be covered with a slight fibrinous layer—such false membranes being liable to form on the surface of any wound in those who are the subjects of diphtheritis. At first, and while the tonsils are covered with this plastic exudation, the symptoms are not severe, so that attention is not directed to the throat. But this is a precious moment for the surgeon: for he may now frequently arrest a disease, which if allowed to go on is usually beyond his art.

While employing the solid caustic, the child should be held by a strong assistant; for it is rare that the practitioner can effect this operation unaided. The tongue must be held down by a very *large spatula*, or by the handle of a *large* spoon. If a small instrument be used you cannot effect your object. We generally employ a large wooden tongue-depressor. The caustic should, for fear of accidents, only project very slightly from its case; and many practitioners, on this account prefer using solutions. We have already said that, at the earliest stage, the use of even a weak solution three times a day suffices. We should apply the caustic beyond the margin of the false membrane as well as to itself, as this will prevent its extension. In serious cases the solution must be very strong (1 part to 3 or 4 water,) but then need only be used once daily. It may be applied by means of sponge fixed at the end of a piece of whalebone by sealing-wax. The caustic frequently dissipates the false membranes upon the amygdalæ, and yet they extend to the epiglottis. Caustic is still our best means. A larger sponge is now required, which must be fixed upon a strong whalebone, bent at an obtuse angle. The surgeon places himself on one side, and introducing the sponge right to the base of the tongue, executes some semi-rotatory movements. Sometimes the epiglottis is raised, and the fragments of false membranes detached from its inferior surface, which may be known by the paroxysm of dyspnœa this gives rise to. The caustic requires to be repeated three or four times in the twenty-four hours.

When, in spite of the energetic use of these means, success does not follow, we must have recourse to *tracheotomy*. M. Guersant has, next to M. Trousseau, performed this operation more frequently than any one else, and speaks unhesitatingly of the propriety of undertaking it, and believes that numerous failures arise from its being too long deferred. When the child will certainly die asphyxiated without, why should we hesitate to open the trachea? for cases have occurred in which the patient has lived, although false membranes have penetrated *even into the larger bronchi*. The vital point which cannot bear the slightest presence of these is the *cordæ vocales*. Practitioners who are aware that a certain number of these cases have proved successful cannot doubt the propriety of operating. M. G. usually employs a straight bistoury, and has several small sponges mounted on whalebone and a curved ring-forceps at hand. If the morbid productions do not reach into the trachea we have only to maintain the aperture patent; while, when they extend lower down, their removal may be attempted by means of the bent forceps. Among the numerous modifications of canula employed to maintain the wound open, that of M. Trousseau is an excellent one. It consists of a *double canula*, so that, when obstruction occurs, the *inner one* may be changed without disturbing that which remains in the wound. This practitioner, as well as M. Bretonneau, prior to introducing the canula, passes small sponges, moistened with a solution of nitrate of silver, into the trachea: but unless false membranes are obviously present, M. G. doubts the propriety of any such interference, and does not have recourse to it. The canula may usually be removed at the end of from eight to twelve days, but sometimes requires to be retained for 20 to 30.

The air of the chamber should not be kept too dry and hot. To render it sufficiently humid it is a good practice to evaporate some emollient decoctions in the room several times a day. It is a difficult thing to maintain an equable temperature about the child, but for a long period I have experienced the utility of wrapping around the neck, without tightening it, a light woollen comforter having its meshes widely knitted. By this contrivance, the air, before it reaches the trachea, becomes sufficiently warmed. When the canula becomes obstructed, the inner canula should be removed and cleansed, instead of thrusting sponges into it, which may only increase the obstruction. When it is deemed proper to cleanse out the trachea, only the most delicate whalebones must be employed. When indurated concretions form, both canulæ should be removed and the patient encouraged to expel them by coughing. I have never removed the canula before the tenth day, but M. Trousseau has done so on the third or fourth. He advises us not to remove it suddenly, but for one, and then for several, hours daily.

Finally, let us observe that croup is so grave and so constantly mortal a disease, that we should frequently have recourse to this operation before it reaches its last stage. "While tracheotomy was almost always a powerless weapon in my hands," says M. Trousseau, "I always recommended its performance as late as possible; but now I have met with numerous instances of success, I always say it should be performed as early as possible, as soon in fact as no other chance of success remains. Of 136 children operated upon, M. T. has saved the lives of 32; and, without being so fortunate as that practitioner, in 36 cases I have met with four successful ones—a success sufficiently great for us to lay it down as a law that we should interfere rather than allow the infant inevitably to die.—*Gazette des Hôpitaux.*

(The practice of promptly examining and cauterizing the pharynx may doubtless prevent the extension of the disease in some cases, and is not enough attended to in this country. So, also, an unreasonable prejudice against *tracheotomy* prevails among us. Death stares us in the face, and why not resort to means which has rescued many lives, albeit these may be few compared to the numbers operated upon—many of whom were *in extremis.* Dr. G. does not notice the external application of heat (to rubefaction) so useful at the outset of croup.)—*Med. Chir. Rev.*

